# Characterization and Implementation of Cocoa Pod Husk as a Reinforcing Agent to Obtain Thermoplastic Starches and Bio-Based Composite Materials

**DOI:** 10.3390/polym16111608

**Published:** 2024-06-06

**Authors:** Andrés Mauricio Holguín Posso, Juan Carlos Macías Silva, Juan Pablo Castañeda Niño, Jose Herminsul Mina Hernandez, Lety del Pilar Fajardo Cabrera de Lima

**Affiliations:** 1 Escuela de Ingeniería de Materiales, Grupo Materiales Compuestos, Universidad del Valle, Calle 13 No. 100-00, Cali 76001, Colombia; holguin.andres@correounivalle.edu.co (A.M.H.P.); juan.macias@correounivalle.edu.co (J.C.M.S.); juan.castaneda.nino@correounivalle.edu.co (J.P.C.N.); 2 Grupo Tribología, Polímeros, Metalurgia de Polvos y Transformaciones de Residuos Sólidos, Universidad del Valle, Calle 13 No. 100-00, Cali 76001, Colombia; letydelpilar.fajardo@correounivalle.edu.co

**Keywords:** thermoplastic starch, bio-based composite material, cocoa pod husk flour, agricultural wastes, composite materials

## Abstract

When the cocoa pod husk (CPH) is used and processed, two types of flour were obtained and can be differentiated by particle size, fine flour (FFCH), and coarse flour (CFCH) and can be used as a possible reinforcement for the development of bio-based composite materials. Each flour was obtained from chopping, drying by forced convection, milling by blades, and sieving using the 100 mesh/bottom according to the Tyler series. Their physicochemical, thermal, and structural characterization made it possible to identify the lower presence of lignin and higher proportions of cellulose and pectin in FFCH. Based on the properties identified in FFCH, it was included in the processing of thermoplastic starch (TPS) from the plantain pulp (*Musa paradisiaca*) and its respective bio-based composite material using plantain peel short fiber (PPSF) as a reinforcing agent using the following sequence of processing techniques: extrusion, internal mixing, and compression molding. The influence of FFCH contributed to the increase in ultimate tensile strength (7.59 MPa) and higher matrix–reinforcement interaction when obtaining the freshly processed composite material (day 0) when compared to the bio-based composite material with higher FCP content (30%) in the absence of FFCH. As for the disadvantages of FFCH, reduced thermal stability (323.57 to 300.47 °C) and losses in ultimate tensile strength (0.73 MPa) and modulus of elasticity (142.53 to 26.17 MPa) during storage progress were identified. In the case of TPS, the strengthening action of FFCH was not evident. Finally, the use of CFCH was not considered for the elaboration of the bio-based composite material because it reached a higher lignin content than FFCH, which was expected to decrease its affinity with the TPS matrix, resulting in lower mechanical properties in the material.

## 1. Introduction

The CPH or “cacota” is an agro-industrial waste derived from the production of cocoa, a tropical crop present in Colombia that contributes to the national economy from the use of the seed, presenting an approximate biomass of 10% of the weight of the fruit [1], while the CPH, is one of the unused and inadequately managed by-products at present, generating foci for the bacterial propagation of Phytophthora spp and being the main cause of economic losses in cocoa activity [2]. 

Cocoa (*Theobroma cacao* L.) is a species native to the Amazon basin region in South America, the weight of its fruit ranges from 0.2 kg to more than 1 kg. The cocoa industry mainly uses the seeds inside the pod husk as the fraction with the highest commercial value for chocolate production. During the pre-processing stages, approximately 52 to 80% of the cocoa fruit is discarded as residual biomass [3,4]. One of the most relevant by-products is its CPH, finding one of its general characteristics, the difficult degradation due to its lignin and cellulose content, where this lignocellulosic material can be used in a wide variety of industrial processes, however, this is generally discarded within the same crops, generating problems such as the proliferation of pathogenic microorganisms [5].

Another of the general characteristics of the CPH consists in the conformation of its structure based on the presence of the epicarp, mesocarp, sclerotic zone, and endocarp, which are plant tissues containing cellulose between 19.7 and 35.0%, hemicellulose between 6.0 and 12.8%, lignin between 14.0 and 38.8%, protein between 5.9 and 10.0%, pectin between 2 and 12.6%, and lipids between 1.5 and 2%. In the pericarp, the presence of lignin, hemicellulose, and ash is highlighted, while in the mesocarp, up to 53% of cellulose can be found as the majority macromolecule, including the existence of pectin, protein, and lipids, the latter, which are also present in the endocarp [4,6,7,8,9]. Considering such composition, a set of unitary operations can be established to take advantage of the lignocellulosic materials present in such by-products for their extraction and subsequent processing to obtain fiber, paper sector products, nanofibers, lignin, biopolymers, rheology modifiers, biocomposites, and biofuels, among other products and raw materials [10]. In the sector focused on the development of new materials, mixtures of starches, pectins, and plasticizers processed using different processing techniques (casting, compression molding, and extrusion) have been reported, achieving adequate processability without resorting to high levels of torque, screw speed, and applied pressure, including increased mechanical properties, despite the increased capacity in water absorption when compared to a pure TPS [11,12,13,14]. Considering the technical value of the cocoa pod husk and the objective of making the most of this by-product, in the present investigation, the CPH was processed to obtain two types of flour with differences in their granulometry, allowing its physicochemical, thermal, and structural characterization to establish the greatest suitability to be used for the development of a bio-based composite material, establishing its mechanical, thermal, and structural characterization.

## 2. Materials

**Figure 1:**
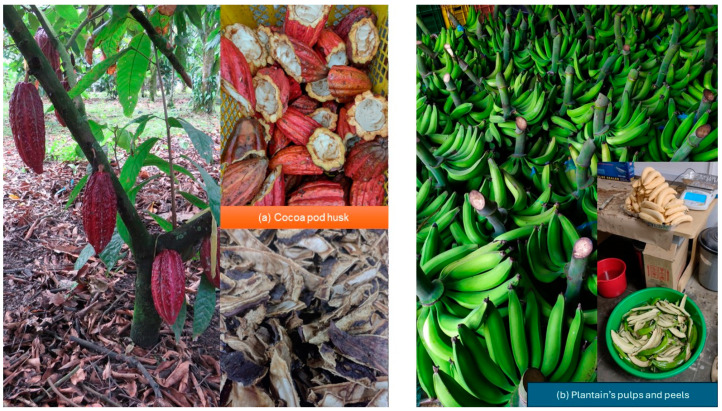
Raw materials for producing bio-based composite material: (**a**) cocoa pod husk; (**b**) plantain pulp and peel variety Dominico hartón.

The cocoa pod husk variety CCN 51 (a by-product of the bean harvest) (see Figure 1a) was supplied by the Asociación de Agricultores de las Veredas Unidas de Caloto (ASOVERUNCA), located in Guachené–Cauca, Colombia, and the plantain variety Dominico hartón was supplied by the Asociación de Productores de Finca Tradicional del Norte del Cauca (ASPROFINCA) (see Figure 1b), located in Villa Rica (Cauca, Colombia). Commercial-grade glycerin was used as a plasticizer and had a polyol of functionality three. Its presentation is a colorless liquid of medium viscosity. Its purity was 99.7%. The plasticizer was supplied by DISAN S.A. (Cali, Colombia).

## 3. Experimental Procedure

### 3.1. Obtaining Plantain Starch

**Figure 2:**
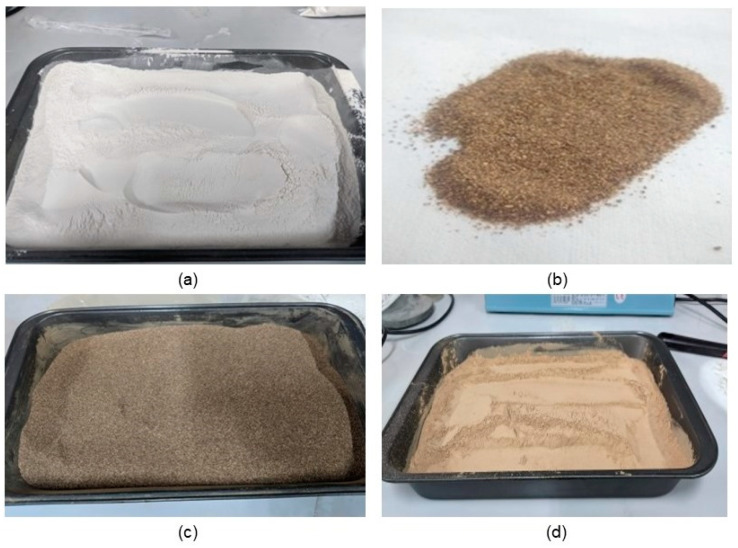
Raw materials are required to obtain bio-based materials. (**a**) Plantain native starch (PNS); (**b**) plantain peel short fiber (PPSF); (**c**) coarse flour from a cocoa pod husk (CFCH); (**d**) fine flour from a cocoa pod husk (FFCH).

Starch was extracted from the pulp of the plantain bunch variety Dominico hartón from the Asociación de Productores de Finca Tradicional del Norte del Cauca (ASPROFINCA) in Villa Rica (Cauca, Colombia). The starch extraction method was based on the use of a 1.2% sodium metabisulfite solution, followed by filtering (separating the slurry and cake), sedimentation of the slurry, drying of the starch paste at 60 °C for 24 h, grinding using a blade mill, and sieving using 100 mesh, according to the Tyler series, presenting a particle size of less than 150 µm. The color of the starch was creamy white (see Figure 2a). Table 1 reports the plantain starch’s thermal, physicochemical, techno-functional, and morphological properties obtained from the pulp.

### 3.2. Plantain Peel Short Fiber (PPSF)

This plantain by-product was obtained by extracting starch from the plantain peel by filtration and separating it from the slurry. The cake resulting from the filtration was then dried in a forced convection oven Binder FD-115 (Tuttlingen, Baden-Württemberg, Germany) at a temperature of 60 °C for 24 h, followed by grinding through a Fritsch cutting mill pulverisette 15 (Idar-Oberstein, Rheinland-Pfalz, Germany) using a sieve with 2 mm openings, and it was then sieved with a 100 mesh/Tyler series bottom, obtaining a particle size of more than 150 µm. The fibers presented a dark green color (see Figure 2b). Other properties identified in this raw material are shown in Table 1.

### 3.3. Obtaining Cocoa Husk Flours

A total of 18 kg of cocoa (*Theobroma cacao* L.) pod husks were collected, sorted, and cleaned using water, detergent, and disinfectant. The material was subjected to size reduction in uniform parts by manual chopping, followed by forced convection drying (80 °C for 24 h) using an oven Binder FD-115. After drying, the resulting CPH chips were introduced into a Fritsch cutting mill pulverisette 15 to obtain the raw meal, passing through a sieve with 0.75 mm openings. Finally, the ground by-product was sieved with the 100/bottom sieve according to the Tyler series, separating the CPH fine flour (FFCH) with a particle size below 150 µm from the CPH coarse flour (CFCH) with a particle size above 150 µm (see Figure 2c,d).

### 3.4. Obtaining Thermoplastic Starch (TPS)

Two types of TPS were manufactured based on the methodology proposed by Mina et al., 2021 [15], differentiating their composition using plantain pulp starch, FFCH, and bio-based composite materials, as indicated in Table 2. Native plantain starch and FFCH should have a water content of less than 6%, whereas the former should be mixed with glycerin at a mixing ratio of 65/35, respectively. One percent stearic acid was added to the resulting mass between starch and glycerin. The mixing was performed using a Kitchen Aid Professional 600 (Benton, MI, USA) mixer, a flat stirrer, and a mixing speed on level 2 for 8 min. Upon completion of the mixing time, the resulting mass was placed in high-barrier plastic bags and sealed for storage for a minimum time of 48 h. Each of the blends was fed into a Thermo Scientific twin-screw extruder, Haake Rheomex CTW 100 Polylab OS (Waltham, MA, USA), to obtain plantain-based TPS pellets using an average temperature profile of 125 °C, a screw speed of 100 rpm, extrusion die for obtaining cord with a 2 mm nozzle, and a barrel with a 25/D ratio. The TPS cord was pelletized and stored in high-barrier plastic bags.

### 3.5. Development of a Bio-Based Composite Material from Plantain TPS and FFCH Reinforced with PPSF

**Figure 3:**
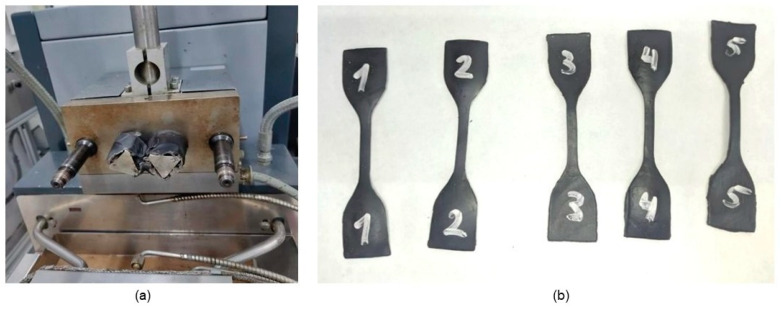
Processing of TPS and bio-based composite material. (**a**) Thermo Scientific internal torque mixer; (**b**) TPS samples made in compression molding (five test specimens).

**Figure 4:**
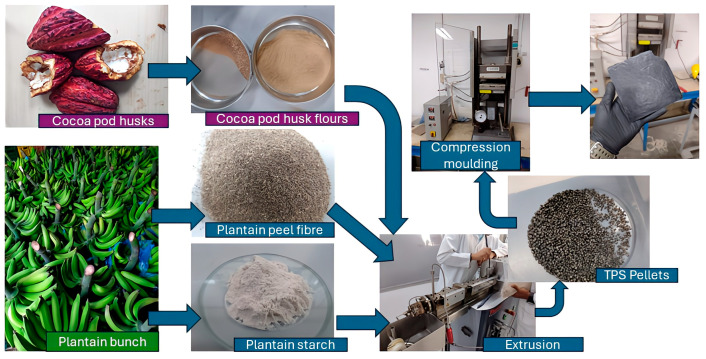
Scheme for obtaining the bio-based composite material from the cocoa pod husk and plantain.

The TPS pellets were introduced into a Thermo Scientific torque mixer, Haake Rheomix (Waltham, MA, USA), and mixed with PPSF (see Figure 3a) using a mixing temperature corresponding to 130 °C, a mixing speed of 50 rpm, and a mixing time of 5 min. The resulting mixtures, subsequently, were destined for compression molding employing a Lab Pro 400 Fontijne Press (Vlambloen, Rotterdam, The Netherlands) with molds in stainless steel, a process temperature of 140 °C, and a pressure of 50 kN for 15 min, followed by a cooling of the resulting sheets, employing thermal shock from water at room temperature [16]. Figure 3b shows the die-cut specimens arranged for mechanical tensile tests. Finally, Figure 4 shows a scheme that summarizes obtaining the bio-based composite material from the cocoa pod husk and plantain.

### 3.6. Thermal, Physicochemical, and Structural Characterization of the Cocoa Pod Husk Flour

#### 3.6.1. Cellulose, Hemicellulose, and Lignin Content

With some modifications, Klason lignin was determined according to Tappi 222- om88 [17]. For this purpose, extractable and water-soluble free fibers (300 mg) were hydrolyzed with 72% (*w*/*w*) sulfuric acid at 30 °C for 1 h in pyrex flasks. Subsequently, the solutions were diluted with distilled water to a 4% sulfuric acid concentration and kept for 1 h at 110 °C. Once cooled, they were filtered through porous glass filters (Vidra FOC 663/3) that were previously tared (4 h at 100 °C). The first 100 mL of the filtrates were collected for subsequent analysis of free sugars. Klason lignin was retained on porous glass filters. The residue (Klason lignin) was washed with distilled water to a neutral pH and dried at 100 °C for 4 h. The percentages of Klason lignin were calculated by weight difference. The ash content of Klason lignin was determined according to Tappi 211 om-85 [18]. Such content was calculated from the total percentage of lignin in the samples [19]. Holocellulose, hemicellulose, and cellulose-α were determined from the Tappi T-203 [20] standard, considering the following equation [21]:

*Hemicellulose* = (*Amount of holocellulose* − *amount of α-cellulose*) (1)

#### 3.6.2. Particle Size

The two types of flour from the CPH previously ground and sieved were subjected to laser particle size analysis according to ISO 13320 [22] in a particle size analyzer with laser beam diffraction technology, a Mastersizer 2000 from Malvern Instruments with a Hydro 2000 MU accessory.

#### 3.6.3. Scanning Electron Microscopy (SEM)

Morphological analysis of the samples was performed on a JEOL model JSM 6490 LV machine (Jeol, Mexico D.F., Mexico). All the samples were coated with a layer of palladium gold to avoid the accumulation of electrical charge during the test using a Denton Vacuum Desk IV cold spray coater model STANDARD A PHENOM (Moorestown, NJ, USA). A total of 1 mg of each flour sample was placed on a carbon ribbon. Also, TPS and the bio-based composite with dimensions of 1 × 3 × 5 cm were used. Initially, each treatment was subjected to immersion in liquid nitrogen for 15 min, followed by fracture by bending to expose and evaluate the fracture cross-sectional area of each treatment. The images obtained were from the backscattered electron method with an acceleration power of 20 kV and a vacuum of 30 Pa in the microscope chamber, achieving magnifications of 250× [23].

#### 3.6.4. Proximal Analysis

This analysis consisted of five basic tests: determining the amount of protein, ash, moisture, fiber, and lipids. Protein determination was carried out according to ISO 1871 [24]. Ash was determined according to ISO 2171 [25]. The moisture percentage was determined according to NTC 529 [26]. Lipids and crude fiber were determined according to NTC 668 [27].

#### 3.6.5. Differential Scanning Calorimetry (DSC)

A total of 10 mg of cocoa flour, TPS, and bio-based composite in each sample was used, previously conditioned at a relative humidity of 50% and a temperature of 23 °C, and evaluated using a TA Instruments Q20 (New Castle, DE, USA) calorimeter. Each of the samples was placed inside an airtight aluminum capsule, sealed, and placed inside the thermal chamber of the DSC. In an inert environment with nitrogen, a first heating cycle from room temperature to 90 °C was performed to clear the thermal history, followed by a 90 °C isotherm for 5 min. This was followed by a cooling cycle from 90 °C to −60 °C and a −60 °C isotherm for 5 min. Finally, a heating cycle from −60 to 250 °C was performed to determine the glass transition temperature (Tg), melting temperature (Tm), and enthalpy (ΔH) in the respective samples. The heating rate was 10 °C/min [23].

#### 3.6.6. Thermogravimetric Analysis (TGA)

Thermogravimetric analysis equipment from TA Instruments, a Q50 (New Castle, DE, USA), was used to study the thermal stability of the flours, TPS, and bio-based composites. The weight of the samples was kept between 5 and 8 mg. Each sample was subjected to a temperature range from 25 to 600 °C at a heating rate of 10 °C/min under a nitrogen atmosphere (analytical grade 5.0) with a heat flow of 50 mL/min. The values obtained are related to the percentage of moisture, weight loss, and decomposition temperature from the derivative of the TGA curve [19,28].

#### 3.6.7. Fourier-Transform Infrared Spectrometry (FT-IR)

An FT-IR spectrophotometer, a Perkin Elmer Spectrum 100 (Bridgeport, CT, USA), was used with an attenuated total reflectance (ATR) accessory. These experiments were performed following ASTM E1252–98 [29]. The samples were kept at a relative humidity of 50% and 23 °C, while the analysis was performed at 100 scans in a range between 4000 and 550 cm^−1^.

#### 3.6.8. X-ray Diffraction (XRD)

In the case of flour, 2 g of material for the elaboration of a pellet was used by compressing it in a rectangular mold at a temperature of 40 °C. For TPS and bio-based composites, the samples were prepared and cut into square sheets with dimensions of 2 cm × 2 cm and a thickness of 3 mm. All the tablets were introduced into the diffraction chamber belonging to the Malvern Panalytical (Jarman Way, Royston, UK) X-ray diffractometer, which was operated at 40 kV using a copper tube (Cukα) for the generation of radiation with a wavelength of 1.54 Å, performing a scan from 2ϴ: 5° to 40° with a step of 0.02° for 10 s. The crystallinity content was determined according to the methodology [30,31].

#### 3.6.9. Tensile Test

Based on ASTM D638 [32], the tension test was carried out using a Tinius Olsen model H50KS (Horsham, PA, USA) universal testing machine employing a type IV specimen. The distance between the grips was 58.25 mm, the speed in the displacement of the moving grip was 5 mm/min, and the load cell capacity was 500 N. The maximum tensile strength (σ_max_), modulus of elasticity (E), and deformation at the breaking point (ε) were determined. Similarly, its tensile properties were monitored during a storage time of 15 days under constant conditions (23 °C and 50% R.H.).

## 4. Results and Discussions

### 4.1. Obtaining Cocoa Husk Flour

In the determination of the dry matter content present in the CPH, a value of 16.59% was identified, which was a high value when compared with Jaimes et al., 2017 [1], who reported values of 6.67 and 10.77% with two drying methods, solar and trays, respectively. On the other hand, using the sequence of unit operations constituted by drying, milling, and sieving to obtain the two types of CPH flours, as shown in Figure 2c,d, FFCH generated a yield of 43.27%. At the same time, CFCH was 54.31%, identifying a loss by milling and sieving of 2.41%. However, if CFCH flour was exposed to a second milling with the same conditions, the share of FFCH increased by 52.0%.

#### 4.1.1. Thermal, Physicochemical, and Structural Characterization of Cocoa Pod Husk Flour

##### Cellulose, Hemicellulose, and Lignin Content

The lignin contents obtained in the two flours are close to those reported by Herrera-Barrios et al., 2022 [33], who found a value of 25.24% in a CPH flour that was crushed and dried at 80 °C and then subjected to milling and sieving to obtain a particle size of less than 150 µm. In the case of the present investigation, using the same unit operations, it was possible to obtain a lower lignin content in FFCH, achieving a value of 20.95 ± 2.0%, while CFCH, which was retained above the 100 mesh according to the Tyler series, had a higher lignin content, presenting a value of 29.83 ± 0.1% (see Table 3). According to the above, lignin and hemicellulose constitute a complex matrix that imbedded cellulose and contributed to generating hardness and mechanical resistance to plant cells for the protection of plant tissues when exposed to mechanical and biochemical stresses [34,35]. The larger size of the particles in CFCH flour possibly contributes to diameters greater than 150 µm when subjected to grinding by blades. On the other hand, a possible greater reinforcing character was identified in FFCH since it had a higher cellulose content and lower hemicellulose content than CFCH, considering the chemical composition identified in the proximal analysis (humidity, ash, protein, and lipids) The contents of cellulose, hemicellulose, and lignin identified in the two flours were similar to what was reported by Bamba et al., 2023 [36].

##### Particle Size

The percentage distribution of the particle size of FFCH and CFCH samples can be visualized in Table 4, which shows the average particle size of FFCH to be 44.04 µm. In comparison, CFCH reported an average of 426.13 µm. According to the above, a significant difference in particle size was found, highlighting the inferiority of FFCH dimensions since the particles comprised between 53 and 250 µm acquire a behavior of greater resistance in their mechanical properties compared to a particle size greater than 250 µm, as reported by Versino, 2017 [37] in his study of biodegradable composite materials with agronomic uses from tuberous roots. On the other hand, considering the previous author, FFCH, with a particle size smaller than 53 µm, can contribute to a reduction in water vapor permeability. The above information can be related to research conducted by Barrios Guzmán et al., 2015 [38], who reported that the particle sizes that recorded higher σ_max_ and E values were between 425 and 600 µm, while a particle size of 1.4 mm provided less effect on reinforcement. 

##### Scanning Electron Microscopy (SEM)

**Figure 5:**
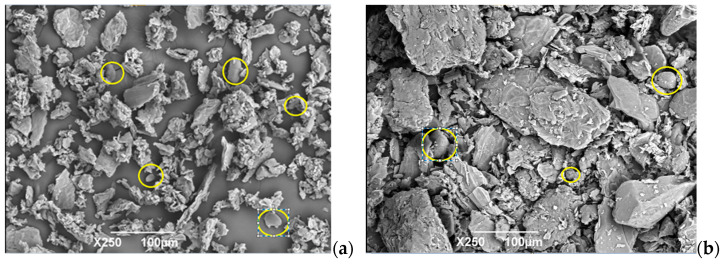
(**a**) FFCH with starch granules; (**b**) CFCH. See the yellow circles in the image.

When observing the micrographs of the flour samples, the presence of cellulosic material and starch granules in a low concentration was identified. The latter component presented the highest proportion in FFCH compared to CFCH, as evidenced in Figure 5. According to Herrera Rengifo et al., 2020 [5], the presence of starch was confirmed at 12.4% (b.h.) in the cocoa husk. In FFCH, particles with smaller dimensions of higher uniformity were observed, while CFCH presented dimensions of higher value and lower uniformity. Considering the above information mentioned by Versino, 2017 [37], FFCH, in terms of dimensions, presents a greater potential for use as a reinforcing agent in bio-based composite materials.

##### Proximal Analysis

The analysis was performed on the two flour samples, identifying moisture, ash, crude fiber, oils, and protein content, as shown in Table 5. When considering the information in Table 5, screening was performed on CPH flour, separating particles smaller and larger than 150 μm. Some components are retained in higher proportions, and a higher ash content was found in FFCH. In contrast, a higher content of crude fiber, lignin, and protein was identified in CFCH. Considering what was obtained by Campos-Vega et al., 2018 [4], CPH flour with an average particle size of 22 μm evidenced a greater reduction in crude fiber and protein content concerning the present investigation. On the other hand, the previous authors mentioned that the type of processing performed on the CPH can alter the values of the results, depending on the unit operations and operation parameters used. Based on the difference found in the ashes between the two flours, a microanalysis by chemical element to X-ray energy dispersive spectra (EDS) was used, finding that the composition of the ashes present in FFCH was made up of carbon (47.14 ± 4.6%), oxygen (37.04 ± 3.4%), magnesium (0.83 ± 0.4%), and potassium (14.97 ± 7.8%). In CFCH, carbon (47.34 ± 1.1%), oxygen (46.07 ± 1.9%), and potassium (6.58 ± 1.9%) were present, corresponding to a percentage in mass. Comparing the results of the research conducted by Peña and Ortega, 2014 [39], in the morphological and structural characterization of fly ash dust obtained from coal combustion and supplied by the Thermoelectric Power Plant Termotasajero S. A. located in the metropolitan area of San José de Cúcuta-Colombia, it was possible to identify the similarity of the elements present in the ashes obtained in the analysis of flours since carbon (53.31%), oxygen (22.12%), magnesium (0.27%), and potassium (0.70%) were found in this research, including other elements such as Na, Al, Si, Ti, Cu, Ca, and Fe that correspond to unburned particles.

##### Differential Scanning Calorimetry (DSC)

**Figure 6:**
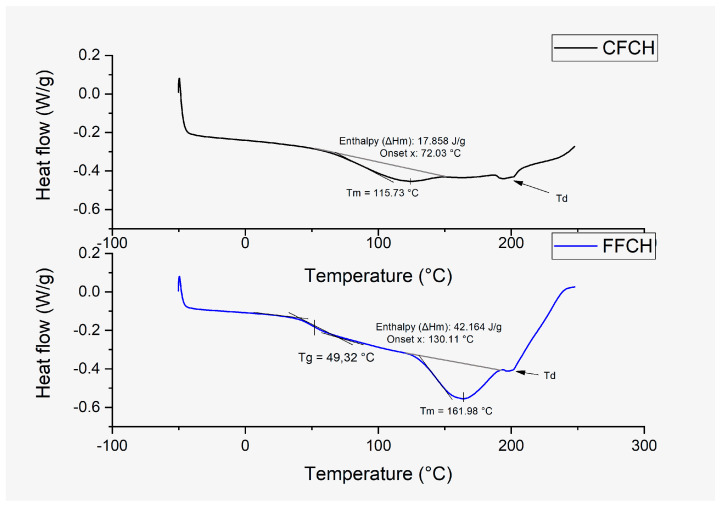
DSC thermograms for the two types of cocoa flour.

Through the analysis of the phase transitions, the melting temperature (Tm) was identified, with its respective enthalpy (ΔHm) and the glass transition temperature (Tg), the latter being a second-order transition (see Figure 6). In the CPH flours analyzed in the present study, these thermal transitions were recorded as follows: FFCH presented a Tm, ΔHm, and Tg with values of 161.8 °C, 42.164 J/g, and 49.32 °C, respectively, while CFCH recorded a Tm and ΔHm of 115.73 °C and 17.86 J/g, respectively. Considering the above results, the presence of a Tg and the higher values presented in the Tm and ΔHm identified in FFCH can be related to the greater presence and availability of pectin after performing the grinding and sieving of the cocoa pod husk since Muñoz Labrador, 2016 [40], identified the Tm in pectins from different sources, molecular parameters, and methoxyl, finding a temperature range between 105 and 157 °C. Based on Tg reports, Seslija et al., 2018 [41], and Kiruthika et al., 2020 [42], reported Tg values of pure pectin between 43 and 50 °C, indicating that the absence of Tg in CFCH relates to its low pectin content.

##### Thermogravimetric Analysis (TGA)

**Figure 7:**
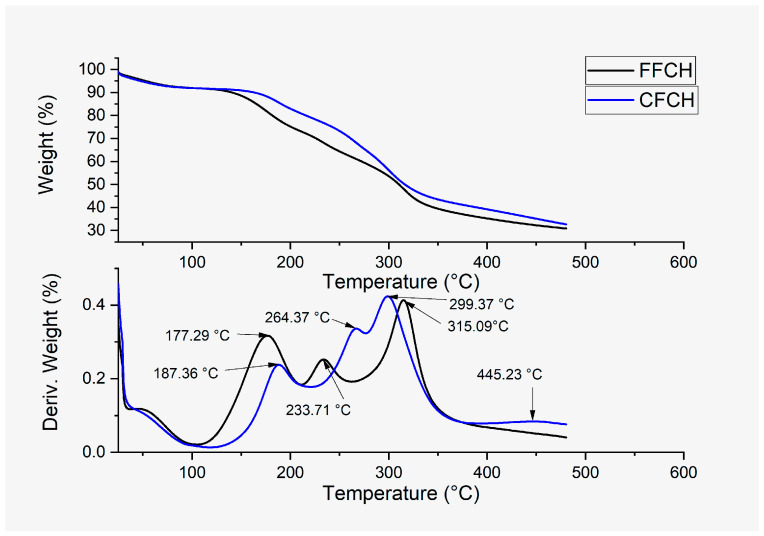
Thermogravimetric analysis of FFCH and CFCH.

CPH flours presented similar thermal behavior from the presence of three peaks in the DTGA, as shown in Figure 7, relating the thermal degradation temperature (Td) of the different macromolecules present in its composition; however, in CFCH, a fourth peak was evidenced at 445.23 °C, relating the decomposition of lignin, as reported by Du et al., 2014 [43], identifying CPH lignin in a temperature range between 300 and 500 °C. In another study, Maache et al., 2017 [44], reported the presence of lignin in native Juncus effusus fibers upon thermal degradation at 455 °C. According to the above, CFCH presented a higher lignin content concerning FFCH and was previously identified by the Klason test. When considering the three signals in common with FFCH and CFCH flours, the first Td peak was recorded at 177.29 and 188.37 °C, respectively, which was attributed to the presence of hemicellulose since Diaz Altamirano and Torres Cabezas, 2019 [45], reported the existence of hemicellulose in a CPH flour by identifying Td in a range between 140.1 and 177.1 °C. The second peak, located at 233.71 and 266.96 °C, respectively, is related to the presence of pectin in the flours, as Bigucci et al., 2008 [46], and Maciel et al., 2015 [47], reported the thermal degradation of pectin to be between 235 and 240 °C. The values of thermal degradation of hemicellulose and pectin at higher temperatures presented in CFCH are possibly due to its larger particle size, requiring more thermal energy to increase the temperature inside the particles that constitute it. On the other hand, when considering the intensities of the peaks representing hemicellulose and pectin, the action of milling by reducing the particle size below 150 µm probably released a higher proportion of hemicellulose with lower interactions with other macromolecules in FFCH, as observed in Figure 7. In contrast, in CFCH, pectin may have presented a higher adhesion or interaction with lignin and/or cellulose since a higher intensity signal was presented in CFCH, possibly relating lignin’s agglomeration capacity within plant structure and the lignin-hemicellulose complex formation [48]. In contrast, the pectin in FFCH may have presented with lower interactions with the other macromolecules. In the last signal identified in the thermograms of FFCH and CFCH, its location was found at 315.09 and 299.04 °C, respectively, attributing the higher Td of FFCH from the possible greater presence of cellulose in a pure or free state (α-cellulose and type I cellulose) since a signal characteristic of lignin was not identified in the flour with a smaller particle size. This is related to what was mentioned by García-Ramón et al., 2021 [49], focusing on the fact that lignocellulosic fibers with a higher cellulose content can generate greater stability in the lignocellulosic fibers.

##### Fourier-Transform Infrared Spectrometry (FTIR)

**Figure 8:**
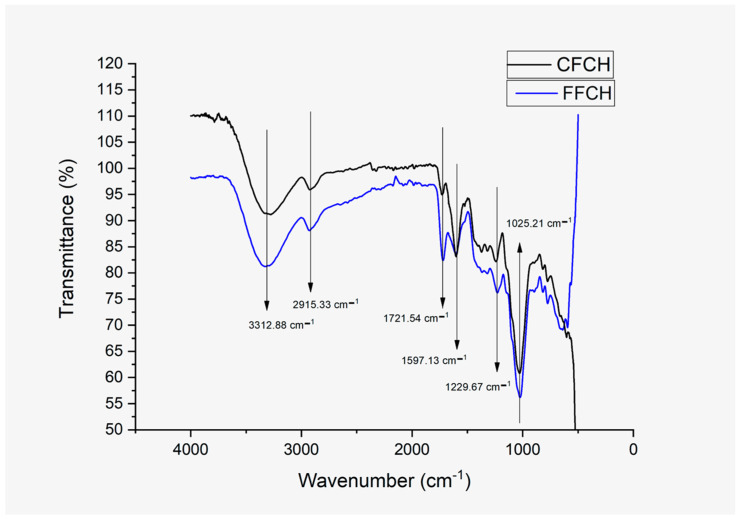
Spectrograms of CPH flour.

In the spectrograms corresponding to the two flours from the CPH (see Figure 8), the same stretching and deformation signals were present relating the presence of O-H bonds (stretching), C-H_2_ (asymmetric stretching), and C=O, C-O-C (stretching), and C-O-H (stretching) bonds [11,50]. The signal identified at 3312 cm^−1^ relates to the O-H groups in cellulose, hemicellulose, and pectin in flours [12,51,52]. The signal located at 2915 cm^−1^ represents the methyl group in the pectin chains and the C-H bonds belonging to the cellulose monomer, glucose [12,52]. The signal identified at 1721 cm^−1^ is related to the stretching of the C=O bonds belonging to the carbonyl ester group (-COO) of pectin, which relates to the degree of esterification of this molecule [11,50,51]. According to what was reported by Prachayawarakorn Jutarat and Pattanasin Worawan, 2016 [50], when making polymer blends with the presence of pectin, the concentration of pectin can be identified according to the intensity of the previously mentioned peak, identifying higher pectin concentration as the intensity of the peak increases. According to the above and what was identified in the DSC, FFCH possibly possesses a higher pectin concentration, as observed previously in Figure 7. The vibrations at 1229 cm^−1^ are related to the ester bonds (C-OO) of pectin and cellulose [12,51], while the signal at 1025 cm^−1^ represents stretching vibrations of C-O-H and C-H_2_ bonds belonging to cellulosic chains [12,36].

##### X-ray Diffraction (XRD)

**Figure 9:**
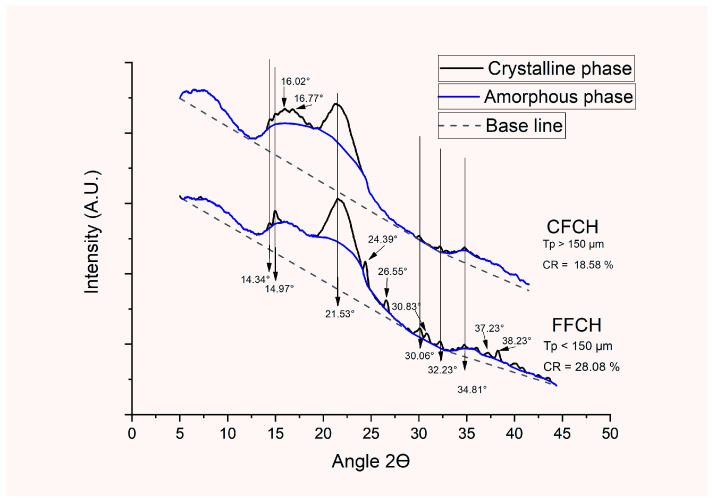
Diffractograms of the CPH flour.

Two macromolecules are found in the CPH flour, pectin, and cellulose, which provide crystallinity in its structure [50,52]. The previously mentioned raw material can contain pectin between 8 and 11% on a dry basis [53]. Other studies, such as the one carried out by Fontes, 1972 [54], obtained a yield of 8.0% of pectin from endocarp on a dry basis, while Suárez and Marín, 2019 [55], obtained a yield of 3.7 and 3.5% in criollo and CCN-51 CPH varieties, respectively, through a process of acid hydrolysis using citric acid as an extracting agent at a pH of 2.5 for 40 min and considering that one of the crystallinity patterns can be related to that granted by the pectin. Moreira et al., 2010 [56], reported a semi-crystalline structure with a high-intensity peak located between 20 and 23°2ϴ in pure pectin from citrus with a degree of methyl esterification of 8.4%. Hernandez-Mendoza et al., 2021 [57], reported a semi-crystalline structure with two amorphous phases and a high-intensity crystallinity peak between 20 and 25°2ϴ in a CPH flour of Mexican origin. In FFCH, 12 crystalline peaks were identified. In comparison, CFCH presented eight peaks, highlighting the presence of a high-intensity peak at 21.53°2ϴ in the two samples, possibly related to the presence of pectin and seven medium-intensity peaks (14, 97, 24.39, 26.55, 30.06, 32.23, 34.81, and 38.23°2ϴ) present in FFCH, which is related to the presence of cellulose. In contrast, the other signals in both samples were low intensity, as observed in Figure 9. Prachayawarakorn Jutarat and Pattanasin Worawan, 2016 [50], identified the crystal structure of pectin from the β-helices located in the diffraction peaks at 18.6, 20.6, and 28.4°2ϴ. However, Adeleye et al., 2022 [51], and Ouattara et al., 2022 [58], mentioned that in the CPH flour, it is possible to superimpose the signal given by pectin at values between 20 and 23°2ϴ with the crystallinity peak of cellulose that can be located between 22.10 and 22.32°2ϴ. Continuing with the crystalline pattern of type I cellulose, Herrera-Barrios et al., 2022 [33], reported a cellulose content of 46.65% in the CPH flour, and the crystalline peaks were distributed between 15 and 17.7, 22, and 26.5, and between 34 and 35°2ϴ [36,51]. Considering the above, FFCH had a higher content of type I cellulose than CFCH despite having contributions of crystallinity from pectin at the time of milling by blades and its respective sieving. On the other hand, considering the methodology of Nara and Komiya, 1983 [31], it was possible to quantify the relative crystallinity in the two types of flours with differences in their granulometry, achieving a relative crystallinity (RC) of 28.08% in FFCH, while CFCH presented a value of 18.58%. Hernández-Mendoza et al., 2021 [57], reported a relative crystallinity of 21.88% in a native CPH flour obtained from dehydration, milling (knife mill), and sieving using meshes with openings between 250 and 600 µm, while Sarmiento-Vásquez et al., 2021 [52], reported a value of 46%.

### 4.2. Production of TPS Based on Plantain and FFCH

#### 4.2.1. Influence of FFCH on TPS and Bio-Based Composite Material

Using physicochemical, thermal, and structural analytical techniques, as well as molecules and macromolecules, in the CPH flour, cellulose, hemicellulose, lignin, pectin, and starch, were identified. When subjected to drying, milling, and sieving, the CPH generated two flours, achieving differences in granulometry, Tm, ΔHm, Td, crystallinity, and intensities in the FT-IR spectrogram signals. According to the above, FFCH presented higher cellulose and pectin content with the lowest average particle size (44.04 µm) and lignin content (20.95%) and could be used as a possible reinforcing agent for the elaboration of TPS and plantain-based composite material reinforced with PPSF. Torres et al., 2019 [59], report the importance of low lignin content in fibers to contribute to the reinforcement of bio-based composite materials.

##### Scanning Electron Microscopy (SEM)

**Figure 10:**
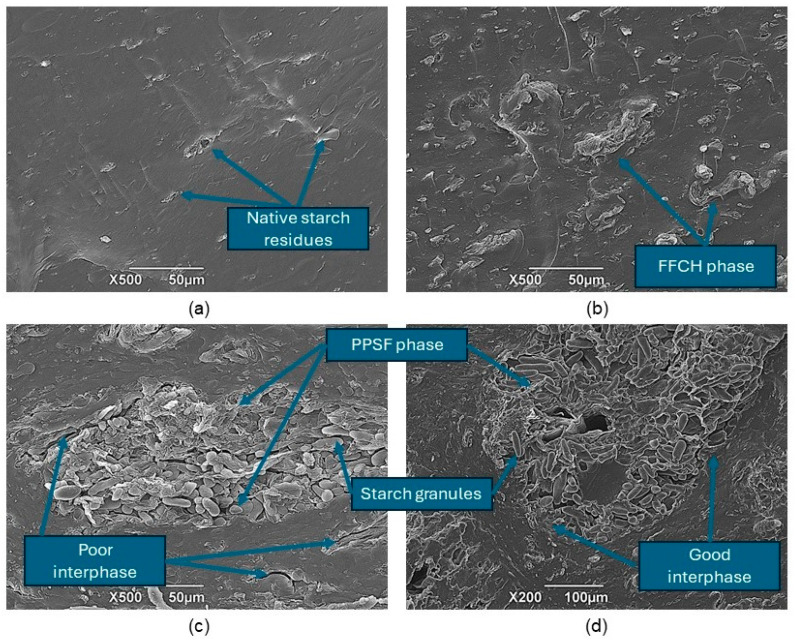
SEM micrographs of (**a**) TPS; (**b**) TPS2; (**c**) TPS + F; (**d**) TPS2 + F, at 200× and 500×.

The micrographs of the treatments can be seen in Figure 10, showing differences in the topography between the two types of TPS and their respective composite materials. When using a magnification of 500×, TPS exposes a low roughness surface that is homogeneous and does not have porosity or cracks, which is characteristic of starch gelatinization; however, plantain starch possesses the “resistant” condition due to its high amylose content [60,61,62]. Residues of starch granules were identified in their native unplasticized state after undergoing the corresponding extrusion profile at 125 °C (see Figure 10a). When FFCH was added to TPS (TPS2), the presence of short fibers was evidenced, relating to the presence of raw fibers in the proximal analysis; on the other hand, an adequate dispersion of the FFCH phase in TPS, a low presence of porosity, and no cracks in TPS-FFCH interfacial phase were observed, relating the possible presence of compatibility between the two components (see Figure 10b). Regarding incorporating PPSF in the two types of TPS, a magnification of 200× was used in their analysis because PPSF presented dimensions between 300 and 550 µm. Another characteristic identified in the reinforcement coming from plantain peel corresponds to the presence of native starch, a residual portion resulting from starch extraction. In TPS + F, low adhesion between the TPS (continuous) and PPSF (dispersed) phases was observed due to the presence of cracks (see Figure 10c). The interface between fiber and starch is not very homogeneous, which can be attributed to the nature of the fibers in which there is the presence of cellulose and lignin, molecules that prevent a good adhesion between the matrix and reinforcement, as reported by Luna and Lizarazo-Marriaga, 2022 [63], in their study of natural fibers as reinforcements in polymer matrix composites; while in TPS2 + F, a lower presence of cracks in the interfacial phase was observed, which is related to the possible interaction of the pectin present in FFCH with the starch polymeric chains (see Figure 10d).

##### X-ray Diffraction (XRD)

**Figure 11:**
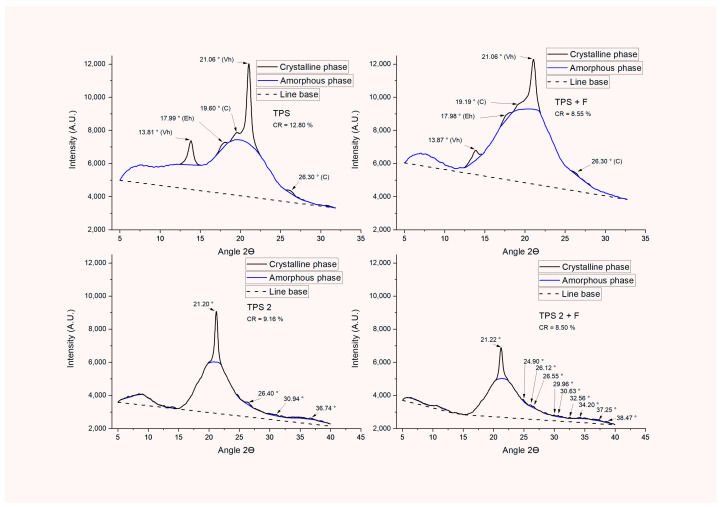
Diffractograms of TPS and bio-based composite materials.

When evaluating the structure of the processed materials, it was possible to define that the residual native crystalline structure type C after processing to obtain TPS, retaining only the peaks 19.6 and 26.3°2ϴ, and in the case of the bio-based compound, these structures were retained at 19.19 and 26.30°2ϴ. Additionally, two crystal structures induced through the interaction between glycerin (plasticizer) and amylose were generated [64], identifying Vh-type crystal patterns constituted by high-intensity peaks between 12 and 14°2ϴ and from 19 to 22°2ϴ [64,65,66,67] and Eh structures comprising medium intensity peaks between 17 and 18°2ϴ [68] (see Figure 11). The incorporation of PPSF contributed to the reduction in the relative crystallinity from 12.80% in TPS to 8.55% in the composite material (TPS + F), possibly due to the increase in the disorder of the starch polymeric chains. For the incorporation of FFCH in TPS, only a high-intensity peak characteristic of induced Vh-type crystallinity was generated at 21.20°2ϴ, a low-intensity peak corresponding to residual native C-type crystallinity at 26.40°2ϴ, and low-intensity peaks starting at 30°2ϴ relating the cellulose coming from FFCH. Despite using raw material, such as FFCH, with an R.C. of 28.08% to produce TPS2, the presence of ordered structures decreased to 9.16%, possibly due to the destruction of the native crystallinity provided by the pectin in FFCH when processed at high temperature and with the presence of shear, contributing to higher amorphous volumes. Although Da Roz et al., 2016 [11], and de Oliveira Begali et al., 2021 [12], reported that the processing of the starch-pectin mixture generates secondary interactions (hydrogen bonds), it did not contribute to new crystalline structures. In the case of obtaining the composite material from TPS2 by adding PPSF, the same behavior evidenced in TPS + F was maintained, including the presence of the low-intensity peaks characteristic of cellulose present in FFCH, which possibly contributed to an additional strengthening.

##### Tensile Test

The mechanical behavior evaluated in the above treatments using the tension test allowed for the identification of the dependence of the σ_max_, the ε, and the E of TPS as a function of storage time (days 0, 8, and 15) and the amounts of FFCH and PPSF (see Table 6). The treatments evaluated on day 0 presented a σ_max_ of 4.09 MPa in TPS, with an ε of 17.33%, while TPS + F obtained values of 5.12 MPa and 1.17%, respectively, relating the presence of reinforcement from 30% of PPSF. According to the above, the mixture of lignocellulosic fibers with TPS contributes to the generation of hydrogen bonds, increasing mechanical properties [69,70]. The incorporation of PPSF in TPS (TPS2) did not contribute to the increase in the σ_max_, generating a value of 3.98 MPa. However, the addition of 15% of PPSF increased the σmax between the composites, presenting a value of 7.59 MPa with an ε of 6.01%, relating the possible interaction between FFCH pectin, TPS, and PPSF, as identified in the SEM. This agrees with what was stated by Prachayawarakorn Jutarat and Pattanasin Worawan, 2016 [50]. When elaborating a bio-based composite material from cassava TPS, commercial-grade pectin with 72% methylation and cotton fibers use an internal mixer followed by compression molding, managing to increase the σmax from 2 MPa in TPS to 14 MPa when elaborating a composite material with 8% pectin. On the other hand, the E corresponding to day 0 in TPS + F and TPS2 + F obtained the highest values, 192.38 and 142.53 MPa, respectively, which relate to a higher bond strength between the bonds that conform them, maintaining the superiority of TPS + F from its higher PPSF content, which is a behavior like that reported by Prachayawarakorn Jutarat and Pattanasin Worawan, 2016 [50]. As storage time progressed, the σ_max_ presented a reduction in all treatments due to the absorption of water generated by its components [49,71]. Both types of TPS reduced their value below 1 MPa due to the greater presence of hydroxyl groups from the starch structure; however, TPS2 presented a more significant reduction in this property since pectin also presents hydroxyl groups in its structure and possibly has greater availability to absorb water as the starch retrogradation progresses [13]. The composite materials reduced the value of the σmax to values between 1.38 and 1.49 MPa on day 8. In contrast, on day 15, values below 1 MPa were generated, indicating that adding PPSF contributed to reducing the water absorption rate in the structure of these treatments. For the ε, the water absorption generated on day 8 in the four treatments contributed to the increase in the capacity to deform, acting as a plasticizing agent and evidencing a higher capacity in the two types of TPS. On day 15, the tensile property was reduced, possibly due to starch retrogradation. Finally, the E showed a reduction in its value in three of the four treatments on day 8, relating the possible decrease in intramolecular interactions of TPS and intermolecular interactions with the fibers and FFCH from the increased absorbed water. In two of the four treatments (TPS and TPS2 + F), and related to what was identified in the ε, the magnitude of the E stabilized and/or increased from the retrogradation of the starch polymeric chains. At the same time, TPS2 continued to reduce its value with the possible greater capacity to absorb water. The value of TPS + F increased due to its probable lower capacity to absorb water and manifest the advance of starch retrogradation, going from 192.38 to 198.65 MPa. At the same time, on day 15, its property was reduced due to the increased absorbed water.

##### Thermogravimetric Analysis (TGA)

**Figure 12:**
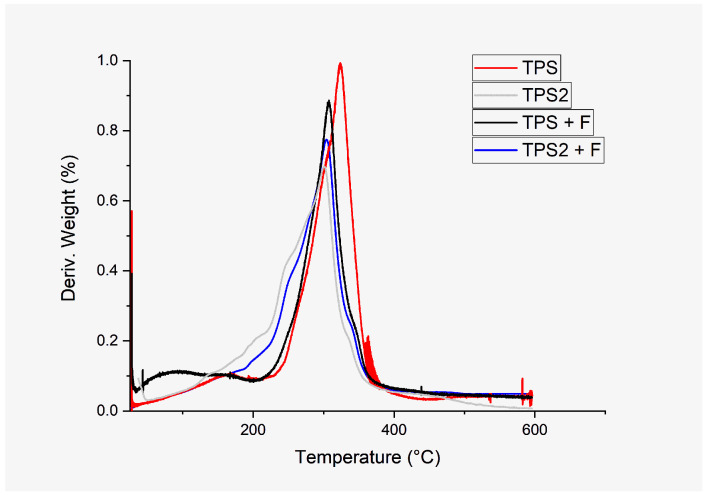
DTGA thermograms of TPS and plantain and cocoa-based composites.

When subjected to high temperatures, TPS presented an onset of thermal degradation (Td) (see Figure 12), corresponding to the high starch content phase at 213.52 °C, reaching a 50% degradation of the starch polymeric chains at 323.57 °C. Bodirlau et al., 2013 [69], Calambás Pulgarin et al., 2022 [72], and Chen et al., 2020 [71], reported the former phase to be between 311 and 335 °C. In the temperature range identified in the treatment, random breaks of covalent or primary bonds belonging to the main polymeric chain of starch were generated [73]. The above values were higher than those obtained for TPS + F, TPS2, and TPS2 + F (see Table 7), indicating that the presence of PPSF and FFCH possibly contain lignocellulosic fibers with an essential portion of hemicellulose and lignin, generating a possible affectation in the formation of hydrogen bonds between starch, glycerin, and lignocellulosic fibers to establish thermal stability [65,74]. García-Ramón et al., 2021 [48], and Zainuddin et al., 2013 [73], similarly concluded that lignocellulosic reinforcements can present greater thermal stability to the extent that their composition presents higher cellulose content and lower lignin and hemicellulose content. TPS2 and TPS2 + F, presented the lowest Td, where possibly the presence of pectin in FFCH presents hydrogen bonds with greater sensitivity to break when exposed to high temperatures since Šešlija et al., 2018 [41], reported the Td of pectin at 240 °C from the DTGA peak, considering that, according to the degree of methoxyl, such temperature can vary between 200 and 320 °C.

##### Differential Scanning Calorimetry (DSC)

The calorimetric analysis of the four treatments was based on the identified changes in the melting temperature (Tm) and enthalpy of fusion (ΔHm). Considering the above, the first-order transition, called the Tm, relates the inter- and intramolecular secondary bonding forces between starch–starch, starch–plasticizer, and starch–fiber [75]. Its endothermic peak is related to the melting of crystals formed from starch [76], evidencing the highest temperature and enthalpy in TPS with a value of 110.16 °C and 330.43 J/g, respectively, concerning the other treatments. By adding PPSF, the thermal properties were reduced, relating to a reduction in the development of the crystalline structure [77]. In the case of TPS2, the Tm and ΔHm continued to be reduced to values of 107.01 °C and 82.65 J/g, evidencing that FFCH does not contribute to the crystalline structure of TPS, which is like the two behaviors previously mentioned, as evidenced by XRD. Meanwhile, TPS2 + F, by incorporating PPSF, increased the thermal properties concerning that was evidenced in its matrix. Although XRD did not evidence an increase in the crystalline phase, the energy of the secondary binding forces between some of the treatment phases (starch, plasticizer, PPSF, and FFCH) was possibly increased since it generated the highest σmax.

## 5. Conclusions

The reason for using FFCH flour for the development of a plantain-based bio-based composite material was based on the smaller particle size (44.04 µm) and higher pectin and cellulose content, as indicated by granulometry, FT-IR, XRD, DSC, and TGA, which achieved greater dispersion of its components in the TPS matrix and provided greater tensile properties. However, it was evident that CFCH flour generated greater thermal stability due to the lower pectin content and greater presence of lignin in its composition. The processing for the preparation of TPS based on plantain and mixed with FFCH flour through twin-screw extrusion contributed to the reduction in torque, which was a related property as a processing aid additive; however, it generated a slightly lower σ_max_ and a greater E concerning the pure plantain TPS, while the incorporation of PPSF in the two types of matrices contributed to an increase in the previously mentioned tension properties, relating an increase in the formation and presence of hydrogen bonds, according to what is indicated in the DSC. However, the TPS2 + F treatment presented the highest magnitude of tensile strength (7.59 MPa) due to the interaction between the plantain starch chains and the pectin from FFCH flour, according to what was evidenced in the SEM. Among the disadvantages of using FFCH flour in the bio-based composite material is the reduction in thermal stability, as identified by the TGA, and the greater speed in the loss of tensile properties (σmax and E) as storage time progresses.

## Figures and Tables

**Table 1:** Thermal, physicochemical, and morphological properties of plantain starch.

Properties	Starch from Plantain Pulp	Fiber from Plantain Peel
Particle size (µm)	13.52 ± 5.21 (Width)	569.96 (average)
	22.90 ± 8.16 (Long)	
Amylose content (%)	28.37 ± 0.28	-
Lignin content (%)	-	22.43 ± 2.50
Gelatinization temperature (°C)	86.50	-
Enthalpy of fusion (J/g)	97.49	224.20
Water absorption index (g _Gel_/g _Sample_)	2.40 ± 0.01	-
Water solubility index (g _Soluble_/g _Sample_)	0.9 ± 0.07	-
Swelling power (g _Gel_/g _Sample_)	2.41 ± 0.01	-
Relative crystallinity (%)	45.78 (C Standard)	-

**Table 2:** Composition of TPS and bio-based composite materials.

Sample	Composition (%)		
	Pulp Starch	FFCH	PPSF
TPS	65.0	-	-
TPS2	50.0	15.0	-
TPS + F ^1^	45.5	-	30.0
TPS2 + F ^2^	42.5	12.7	15.0

The fiber content of the plantain peel ^1^ is 30% and ^2^ 15%. The difference in each treatment corresponds to the plasticizer.

**Table 3:** Cellulose, hemicellulose, and lignin content.

Type of Flour	Holocellulose (%)	Cellulose (%)	Hemicellulose (%)	Lignin (%)
CFCH	32.08	16.20 ± 0.99	15.88 ± 3.01	29.83 ± 0.10
FFCH	36.35	25.02 ± 2.18	11.33 ± 0.43	20.95 ± 2.00

Mean ± standard deviation.

**Table 4:** Average percentage distribution of particle size for the different flour samples.

Sample	d (0.1) µm	d (0.5) µm	d (0.9) µm	D (3.4) µm
FFCH	4.549	23.393	116.217	44.038
CFCH	217.384	410.913	679.593	426.131

Percentile d (0.1): The particle size below which 10% of the sample remains. Percentile d (0.5): The particle size at which 50% of the sample is below and 50% is above. Percentile d (0.9): The particle size below which 90% of the sample remains. D [3,4]: The mean diameter of the distribution, considered in volume.

**Table 5:** FFCH and CFCH flour physicochemical properties.

Chemical Composition	Type of CPH Flour (%)		
	FFCH (<150 µm)	CFCH (>150 µm)	Campos-Vega et al., 2018 (22 µm) [4]
Humidity (%)	6.2	5.5	10.5
Ash (%)	24.0	22.4	9
Crude fibre (%)	38.1	47.2	36.6
Lipids (%)	<5.0	<5.0	1.5
Protein (%)	<2.0	3.69	2.1

**Table 6:** Tensile test results for TPS mixtures.

Sample	Day	σ_max_ (MPa)	E (MPa)	ε (%)
TPS	0	4.09 ± 0.14	56.28 ± 21.83	17.33 ± 2.03
	8	0.78 ± 0.12	6.69 ± 1.60	36.02 ± 7.96
	15	0.75 ± 0.10	6.70 ± 1.04	26.79 ± 7.71
TPS + F	0	5.12 ± 1.07	192.38 ± 34.00	1.17 ± 0.60
	8	1.49 ± 0.16	198.65 ± 71.89	15.53 ± 2.20
	15	0.90 ± 0.20	92.19 ± 27.46	11.22 ± 2.90
TPS2	0	3.98 ± 0.83	76.01 ± 23.93	9.09 ± 1.00
	8	0.67 ± 0.07	9.96 ± 2.98	21.20 ± 1.26
	15	0.57 ± 0.07	7.90 ± 3.09	14,05 ± 0.67
TPS2 + F	0	7.59 ± 0.66	142.53 ± 13.00	6.01 ± 0.11
	8	1.38 ± 0.20	21.86 ± 10.93	13.30 ± 4.17
	15	0.73 ± 0.08	26.17 ± 7.33	10.23 ± 2.96

Mean ± standard deviation.

**Table 7:** Thermal results of TPS mixtures.

Sample	T_m_ (°C)	ΔH_m_ (J/g)	Td_onset_ (°C)	Td_peak_ (°C)
TPS	110.16 148.14	330.43	213.52	323.57
TPS + F	101.59	301.75	206.58	307.37
TPS2	107.10	82.65	184.87	300.47
TPS2 + F	92.28	179.28	187.03	304.41

## Data Availability

Data are contained within the article.
